# Bio-Catalysis for the Functionalization of Cellulose Nanocrystals

**DOI:** 10.3390/nano12224064

**Published:** 2022-11-18

**Authors:** Laura Peponi, Karla A. Barrera-Rivera, José M. Kenny, Ángel Marcos-Fernandez, Antonio Martinez-Richa, Daniel López

**Affiliations:** 1 Instituto de Ciencia y Tecnología de Polímeros, ICTP-CSIC., Calle Juan de la Cierva 3, 28006 Madrid, Spain; 2 Departamento de Química, Universidad de Guanajuato, Noria Alta s/n, Guanajuato 36050, Guanajuato, Mexico

**Keywords:** bio-catalysis, enzyme, covalent functionalization, cellulose nanocrystals, green chemistry

## Abstract

In this work, the chemical modification of cellulose nanocrystals (NCs) using an enzyme as a catalyst has been performed by a “grafting from” reaction, in order to covalently functionalize the external surface of NCs with both poly(L-lactic acid) (PLLA) and poly(ε-caprolactone) (PCL) by ring-opening polymerization. Firstly, cellulose nanocrystals were prepared from commercial cellulose microcrystals by acid hydrolysis and then functionalized by using *Yarrowia lipolytica lipase* immobilized on Lewatit resin as a catalyst. To confirm the success of the grafting reactions, ^1^H-NMR has been performed as well as FT-IR and Raman spectroscopy. Moreover, thermogravimetric analysis has been used to determine the amount of polymeric chains grafted onto the surface of cellulose nanocrystals. Furthermore, the crystalline nature of the polymeric chains grafted onto the cellulose surface has been studied by DSC, X-ray scattering, as well as SAXS analysis. To our knowledge, it is the first time that a biocatalyst approach has been used to obtain biopolymeric functionalized cellulose nanocrystals.

## 1. Introduction

Bio-catalysis is an emerging research area with both substantial scientific and technological interests, as well as a remarkable impact on environmental issues considering the emerged principles for the circular economy [1,2,3,4]. The capability to graft synthetic polymers onto carbohydrates is interesting for obtaining new materials’ applications, such as detergents, packaging, and pharmaceuticals, among others [5,6,7]. From an environmental point of view, this approach can lead to an increment in the biodegradability of polyesters [7]. However, selective functionalization of carbohydrates is a challenging task to accomplish considering that carbohydrates contain a variety of hydroxyl groups with different chemical reactivities [8,9,10]. In general, enzymes are highly selective catalysts, being used to region-selectively acylate carbohydrates [11,12]. In this regard, the synthesis of polyester based on the lipase-catalyzed reaction can be considered an attractive alternative to poorly selective chemical catalysts [13,14,15,16]. Among them, immobilized *Yarrowia lipolytica lipase* (YLL) has proved to be useful for bulk ring-opening polymerization of PCL in the presence of isosorbide [13,17,18,19].

In this work, a bio-catalytic approach for the modification of covalently functionalized cellulose nanocrystals is reported. To our knowledge, it is the first time that the grafting reaction onto the surface of the cellulose nanocrystals has been reported by using both biocatalysts as well as biopolymers to functionalize them. In fact, in the scientific literature, the functionalization of the cellulose nanocrystals with biopolymers has been reported, but a common catalyst has always been used [8,19,20,21]. Here, the covalently functionalized cellulose nanocrystals have been modified by grafting biopolymeric chains onto the external surface of the cellulose nanocrystals by using an enzyme as a biocatalyst. Therefore, an environmental-friendly approach has been used to increase the compatibility of hydrophilic cellulose nanocrystals with hydrophobic biopolymeric materials for further applications in bio-nanocomposites. 

## 2. Materials and Methods

### 2.1. Materials 

Microcrystalline cellulose, L-lactide (L-LA), ε-caprolactone (CL), and dry toluene were purchased from Sigma Aldrich. All materials were used without further purification.

Cellulose nanocrystals (NCs) were synthesized from commercial microcrystalline cellulose by acid hydrolysis following the same methodology reported by us in previous works [22,23].

*Yarrowia lipolytica lipase* (YLL) has been used as a catalyst for the grafting reaction in order to functionalize the cellulose nanocrystals. The enzyme was previously immobilized into a resin, as reported by us [13,17,18]. Lipase activity was measured using the simplified method of the para-nitrophenyl palmitate assay for lipases and esterases previously described by Gupta et al. [24]. One unit of enzyme activity is defined as the amount of enzyme liberating 1 μmol of p-nitrophenol per minute. As we reported previously [25], immobilized lipase on Lewatit VPOC-1026 has an activity of 47 U/g.

Functionalization of cellulose nanocrystals: The surface modification of cellulose nanocrystals was performed by a “grafting from” reaction onto the cellulose surface by ring-opening polymerization (ROP) of L-lactide or ε-caprolactone, using superficial hydroxyl groups as initiators as well as YLL as a biocatalyst. The reaction conditions used were the same in both cases. Briefly, dried cellulose nanocrystals, previously synthesized, were dispersed in dry toluene by using an ultrasonic bath for 90 min. Then, 10 mmol of monomer was dissolved in dry toluene for 15 min at 50 °C and then added to the cellulose-toluene solution. Immobilized enzyme was added as a biocatalyst (2:1 mg/mmol with respect to the monomer) and the reaction was heated at 90 °C for 7 days. When the reaction finished, to remove the unreacted monomer as well as the non-grafted polymer and the immobilized enzyme, the reaction was filtered and precipitated in cold methanol and then dissolved in chloroform. 

Under the same experimental conditions, but without cellulose nanocrystals, both neat PCL and PLLA were synthesized to compare their polymerization processes with respect to the polymerization onto the surface of the NCs. 

### 2.2. Characterization

The morphology of cellulose nanocrystals was studied by transmission electron microscopy (TEM), by using a JEOL JEM-2100 TEM instrument (JEOL Ltd., Akishima, Tokyo, Japan), with the LaB6 filament and with an operating voltage of 200 kV. For the TEM analysis, the solutions were directly cast onto the 200-mesh cooper grid followed by solvent evaporation at ambient conditions for 24 h.

Solution 1H NMR spectra were recorded at room temperature in a Varian Unity Plus 300 instrument (Palo Alto, CA, USA) using deuterated chloroform (CDCl3) as a solvent. Spectra were referenced to the residual solvent protons at 7.26 ppm.

Fourier transform infrared spectroscopy (FT-IR) measurements were conducted by a Spectrum One FT-IR spectrometer (Perkin Elmer instruments). Spectra were obtained in the 4000–400 cm^−1^ region at room temperature in transmission mode with a resolution of 4 cm^−1^.

Raman spectra were obtained using a Renishaw InVia Reflex Raman system (Renishaw Ibérica, S.A.U., Barcelona, Spain). An optical microscope was coupled to the system. The Raman scattering was excited using a di-ode laser at a wavelength of 785 nm. The laser beam was focused on the sample with a 100 × 0.85 microscope objective. The laser power was 350 mW. The exposure was 10 s and there were 10 accumulations for the Raman measurements. The Raman spectra were taken at different positions of the sample, obtaining the same spectrum, indicating that the sample was homogenous.

Thermogravimetric analysis (TGA) was carried out using a TA-TGA Q500 instrument (TA Instruments, New Castle, DE, USA). The experiments were performed on samples weighing around 10 mg from room temperature to 700 °C at 10 °C/min under nitrogen atmosphere with a flow of 60 mL/min. 

Differential scanning calorimetry (DSC) was performed in a Mettler Toledo DSC822e instrument (Mettler-Toledo, Schwarzenbach, Switzerland). Samples were sealed in aluminum pans. Two scans (0–200 and −90–200 °C) were performed with a heating rate of 10 °C/min and a cooling rate of −10 °C/min between runs under nitrogen purge. The melting point (Tm) is given as the maximum of the endothermic peak. Data reported were taken from the second scan. 

Wide-angle X-ray diffraction (WAXD) measurements were performed using a Bruker D8 Advance instrument (Bruker, Madrid, Spain) with a Cu Kα source (0.154 nm) and a detector Vantec1. The scanning range was 2°–50°, and the step size and count time per step were 0.023851° and 0.5 s, respectively.

SAXS measurements were performed at the ALBA synchrotron facility (Cerdaenyola del Vallès, Spain). Samples were sealed in DSC aluminum pans, placed within a Linkam hot stage, and heated at 10 °C/min while the SAXS spectra were recorded. Calibration of temperature yielded a difference of approximately 7 °C between the temperature reading at the hot stage display and the real temperature of the sample. The detector distance was 6.3296 m, and the detector calibration was performed with the rat tail tendon. The long period, L, was calculated as 2π/q, where q is the scattering vector, from the representation Iq^2^ vs. q. The representation Iq^2^ vs. q facilitates the detection of the maximum, especially when using automated macros for the processing of the high amount of data generated in a synchrotron experiment. Moreover, it is well-known from the scientific literature that the long period, L, can be calculated directly from the curve Iq^2^ vs. q when the polymer presents a lamellar morphology, as is the case of PCL [26,27,28].

## 3. Results

**Figure 1:**
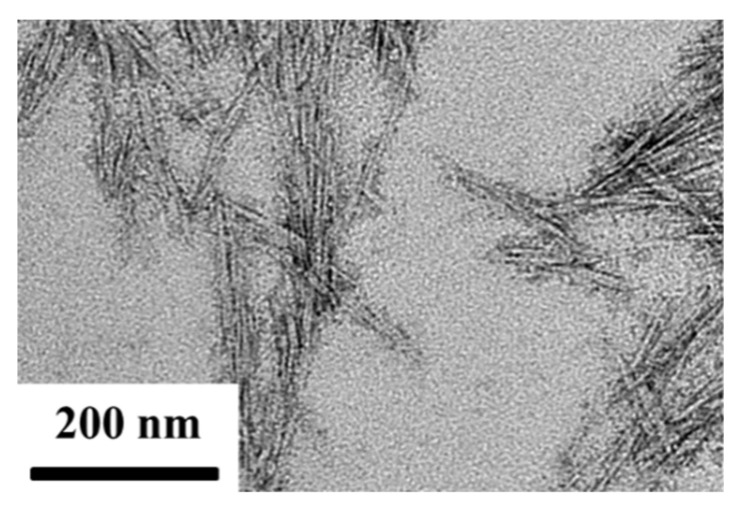
TEM image of synthesized cellulose nanocrystals.

A TEM image of cellulose nanocrystals (NCs) synthesized by acid hydrolysis in our labs is shown in Figure 1. 

From Figure 1, it is easy to note the characteristic rod-like shape of NCs, with a diameter of about 17 ± 2.5 nm and a length of about 170 ± 40 nm. These values are in agreement with those described in the scientific literature [21,29]. 

**Scheme 1:**
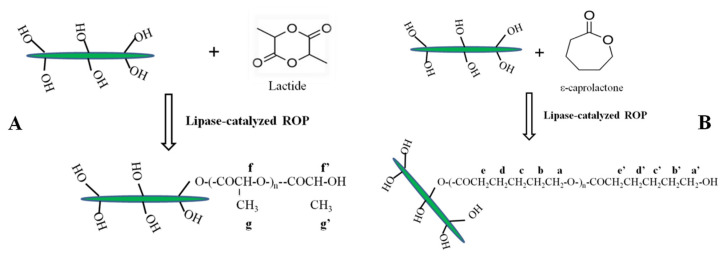
Scheme of the green catalytic approach for the functionalization of the cellulose nanocrystals with PLLA chains (**A**) and PCL chains (**B**) grafted onto the external surface of the cellulose nanocrystals.

Surface NCs’ hydroxyl groups were used as initiators for the grafting reaction of both polyester chains by using lipase as a catalyst to obtain nanocrystals functionalized with PLLA and PCL chains, named NC-g-PLLA and NC-g-PCL, respectively, as indicated in Scheme 1.

**Figure 2:**
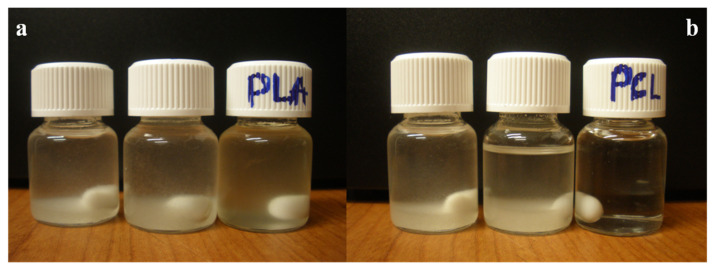
Solutions in chloroform, after 30 min of settling, for (from left to right) pristine cellulose nanocrystals, mixture of cellulose nanocrystals and homopolymer, and grafted cellulose nanocrystals (**a**) with PLLA and (**b**) with PCL.

As it is well-known, cellulose is a hydrophilic polymer; consequently, the dispersion of unmodified cellulose in organic solvents is poor [8,30]. Once functionalized, both PLLA- and PCL-modified cellulose nanocrystals form stable suspensions in chloroform, as shown in Figure 2. In both photographs, three different solutions were prepared with the same amounts of cellulose nanocrystals in chloroform. The solutions were stirred for 30 min and the pictures were taken after 30 min of settling. In both pictures, the first vial contains only cellulose nanocrystals, the second one contains a mixture of NCs with PLLA or PCL (Figure 2a,b, respectively), and the last one contains the modified cellulose nanocrystals. It can be seen that only the vials containing NC-g-PLLA in Figure 2a, and NC-g-PCL in Figure 2b, are stable. In fact, in the case of modified cellulose nanocrystals, it is easy to note no decantation of cellulose nanocrystals at the bottom of the vials, thus confirming that the grafted reaction modified the hydrophilic nature of the cellulose surface, improving the cellulose nanocrystals’ dispersion in an organic solvent. This is clear evidence, commonly used in the scientific literature [8,20,22] to visualize the results for a well-done functionalization. The only difference we obtained between the two different modifications is that in the case of NC-g-PCL, a transparent solution was obtained, while for NC-g-PLLA, a translucent one was observed. 

**Figure 3:**
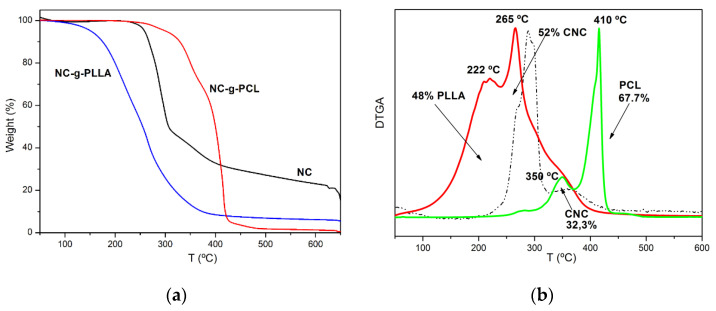
Weight loss curves (**a**) and DTGA thermograms (**b**) for: neat cellulose NC (black), NC-g-PCL (green), and NC-g-PLLA (red).

From the TGA, it is possible to calculate the amount of both cellulose nanocrystals and grafted polymeric chains [22]. In this regard, in Figure 3a the weight loss curves for neat and modified cellulose nanocrystals are represented. The derivative of weight loss for both NC-g-PCL and NC-g-PLLA is reported in Figure 3b. In both cases, two overlapped peaks can be observed, which correspond to the thermal degradation of cellulose nanocrystals as well as to those specific for the polymeric chains. Therefore, the amounts of the different components were calculated by fitting the curves with two Gaussian functions. As we expected, due to the very short chains of PLLA grafted onto the surface of the cellulose nanocrystals, the first degradation peak for NC-g-PLLA corresponded to the thermal degradation of the PLLA chains, at about 222 °C, while cellulose nanocrystals presented a maximum degradation rate corresponding to 265 °C. In this case, the amount of PLLA grafted chains was about 48 wt.%. 

These results are in good agreement with the results already reported in the scientific literature. For example, we previously reported that in the case of cellulose nanocrystals grafted with PLLA chains by a traditional method, such as using the traditional catalyst stannous octoate, the CNC degradation peaks present a maximum corresponding to 266 °C, while PLLA shows the maximum peak at 219 °C [22].

For NC-g-PCL, the first peak corresponded to the degradation of the NCs at about 350 °C, while the maximum rate of degradation for the PCL grafted chains was about 410 °C. It was also found that the amount of grafted PCL was about 68%. From Figure 3, it is clear that both grafting reactions strongly modified the degradation behavior of neat NC. For NC-g-PLLA, the degradation started at lower temperatures, while for NC-g-PCL the degradation proceeded at higher temperatures with respect to neat cellulose nanocrystals. These values are in agreement with those already reported in the scientific literature for NC-g-PCL by using a traditional catalyst such as stannous octoate [7].

**Figure 4:**
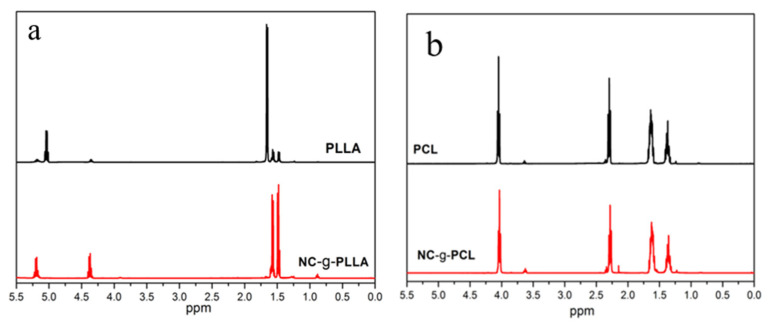
^1^H-NMR spectra for: PLLA and NC-g-PLLA (**a**) and PCL and NC-g-PCL (**b**) synthesized under the same experimental conditions.

However, to confirm the success of the grafting reactions, ^1^H-NMR experiments were performed and reported in Figure 4. In Figure 4a, the ^1^H-NMR spectra for PLLA and NC-g-PLLA, and in Figure 4b the spectra for PCL and NC-g-PCL synthesized under the same experimental conditions, are shown. 

Regarding the polymerized PLLA (see Figure 4a), a large amount of unreacted LA (signal at 5.03 ppm) was observed, while NC-g-PLLA did not show signals of unreacted LA at the same experimental conditions. In both cases, PLLA and NC-g-PLLA, the multiplet at 5.05–5.25 ppm corresponds to methine protons of polymerized lactide (f) and the signal at approximately 4.35 ppm corresponds to the terminal LA units (f′). However, in the case of the PLLA chains grafted on the surface of the cellulose nanocrystals, a very small molecular weight, calculated as 155 Da by using the ratio of the integrals for end groups and internal LA units, has been obtained. This fact indicates that only one lactide unit has reacted with cellulose hydroxyl groups. In particular, considering the amount of NC and PLLA chains in the modified materials, NC-g-PLLA, obtained by TGA calculation, afforded an almost complete reaction of the primary hydroxyl groups of the cellulose nanocrystals, that is 96%. Therefore, when the primary hydroxyl groups of the cellulose nanocrystals open, the LA units generate secondary hydroxyl groups that are not able to open the lactide ring, as occurred for the secondary hydroxyl groups of cellulose nanocrystals. PLLA chains grafted onto the NCs’ surface are very short: only one lactide unit (two lactic acid molecules) was formed, clearly influencing their crystalline nature. Moreover, the doublets at 1.4–1.7 ppm correspond to the LA methyl protons g and g′, corresponding to the unit attached to the cellulose and the terminal unit, respectively (see Scheme 1). The obtention of short-grafted chains seems to indicate that only primary hydroxyl groups of the cellulose are able to react to open the lactone ring, and when only secondary hydroxyl groups are presented, the ROP reaction stops. 

In the case of PCL and NC-g-PCL, Figure 4b, it can be noted that long PCL chains were produced and the characteristic signals [26] for polymerized caprolactone were observed. In fact, the triplet at 4.05 ppm is associated to proton *a* from the CL unit linked to another CL unit, and the triplet at 3.74 ppm to proton *a′* from the CL terminal. The triplet at 2.30 ppm is related to proton *e* from CL units linked to other CL units. The multiplet at 1.66 ppm is related to protons *b* and *d,* while the multiplet at 1.39 ppm is related to proton *c*. No rests of unpolymerized caprolactone were detected at 2.63 ppm. From the integrals of the signals, the molecular weight of the polymeric PCL chains was determined by the ratio of the signal *a* of the internal CL units to the signal *a′* of the terminal CL units. We obtained for the polymerized PCL, probably initiated by residual water, a molecular weight of about 4560 Da, and for the NC-g-PCL a molecular weight of about 1920 Da. As expected, the length of the polymerized PCL and the length of the PCL chain grafted onto the surface of the cellulose nanocrystals were quite different. For PCL, initiation is produced by spurious water in the reaction medium, whereas for NC-g-PCL the initiation is performed by the primary hydroxyl groups on the cellulose surface, as already seen for PLLA. From the previous results obtained by TGA and 1H-NMR analysis, we obtained that only 18% of the primary hydroxyl groups of cellulose nanocrystals have reacted with CL units. This fact seems to indicate that once the CL unit is opened, the primary hydroxyl group generated is more reactive than the primary hydroxyl groups of cellulose, indicating that in the case of PCL functionalization, the PCL chain growth is advantaged with respect to the initiation by the primary hydroxyl group on the cellulose. 

**Scheme 2:**
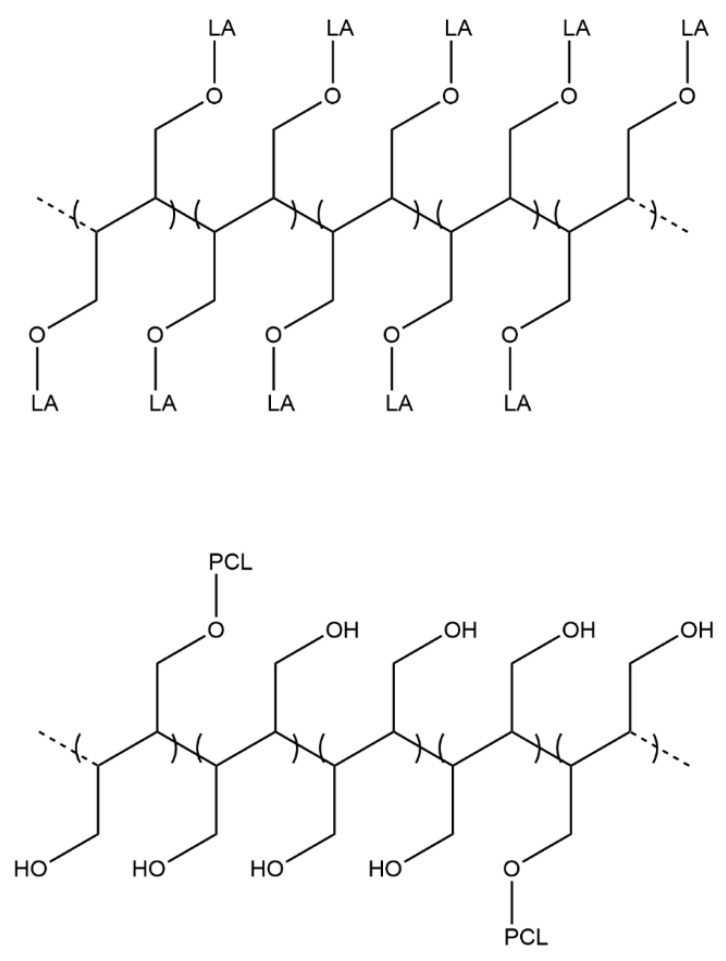
Scheme of NC-g-PLLA and NC-g-PCL.

To facilitate the comprehension of this reaction, in Scheme 2 a scheme indicating only the primary hydroxyl groups of cellulose is reported. In the case of NC-g-PLLA, all the cellulose primary hydroxyl groups have reacted with LA units, while for NC-g-PCL, only 1 in 5 primary hydroxyl groups reacted with CL units, obtaining long PCL chains. 

The fact that a very short LA unit is grafted on the surface of cellulose can also explain the obtention of the translucent solution in Figure 2. 

**Figure 5:**
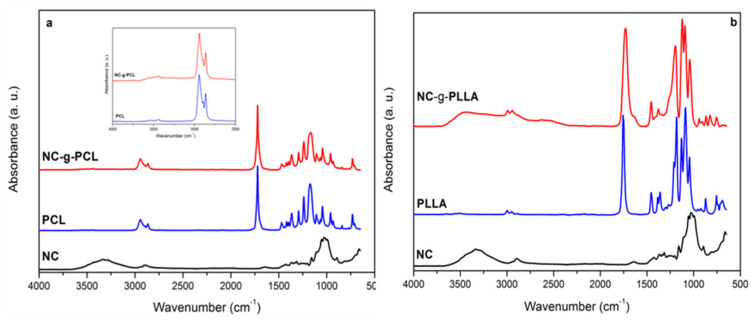
FT-IR spectra for: neat cellulose NC, PCL, and NC-g-PCL (**a**), and NC, PLLA, and NC-g-PLLA (**b**).

**Figure 6:**
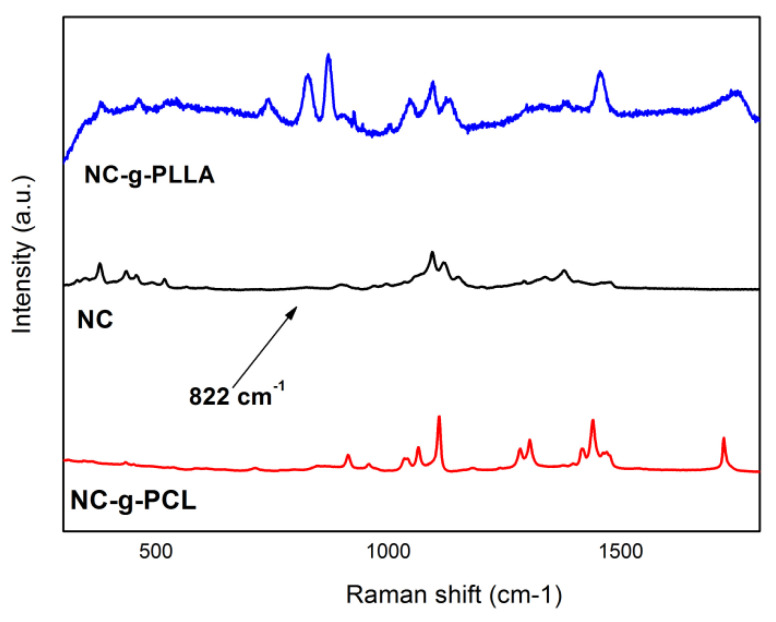
Raman spectra for: neat cellulose NC, NC-g-PCL, and NC-g-PLLA.

The vibrational spectroscopy measurements can be considered another powerful technique to characterize the functionalization reaction. In this work, FT-IR (Figure 5) and Raman spectra (Figure 6) have been recorded. 

In Figure 5a,b, the spectra for the functionalized NC obtained by grafting PCL and PLLA, respectively, onto the external surface of the cellulose nanocrystals are shown. The presence of characteristic peaks is evident for both the NC and the polymeric chain. For PCL, in the inset of Figure 4a, the range 3000–4000 cm^−1^ is magnified to appreciate its peaks. The characteristic stretching band for hydroxyl groups in the region of 3000 to 3600 cm^−1^ can be observed in both cellulose-grafted NC spectra. In fact, the broad peak of hydroxyl groups of cellulose NC from 3200 to 3600 cm^−1^ and the presence of the sharper absorption of the carboxyl group of PCL at 1721 cm^−1^ reflect the presence of both components in the NC-g-PCL sample (Figure 5a). Similarly, the band of the PLLA carbonyl group at 1753 cm^−1^ was found in the NC-g-PLLA spectrum (Figure 5b). 

From the Raman spectra (Figure 6), the presence of both cellulose NC and PCL or PLLA is also demonstrated by the appearance of the characteristic peaks for both in the NC-g-PCL or NC-g-PLLA spectra. 

In particular, the characteristic bands for cellulose, such as the bands between 1200 and 1450 cm^−1^ due to C–C-H bending, CH_2_ rocking and wagging, and C–O-H bending modes, were detected in our spectra. The cellulose crystallinity can also be estimated from its vibration spectra, considering that the 380 cm^−1^ peak was observed only for crystalline cellulose [7]. At the same time, the most relevant characteristic PCL peaks at 912, 1109, 1303, and 1443 cm^−1^ have been detected, also indicating the crystalline nature of the PCL chains [29]. Moreover, the narrow peak at 1720 cm^−1^ was assigned to C=O stretching mode in NC-g-PCL [7]. For the PLLA chains, the most relevant peaks can be summarized as follows: 873 cm^−1^ assigned to C-C stretching and around 1760 cm^−1^ assigned to the carbonyl vibration. 

The amorphous/crystalline nature of the synthesized materials was tested by several techniques. Only the PCL and the NC-g-PCL showed a crystalline nature, with a melting temperature of 55 and 50 °C, respectively (Table 1), as expected considering their calculated molecular weight. Moreover, considering the amount of PCL grafted onto the surface of the cellulose nanocrystals, the degree of crystallinity for NC-g-PCL and for the synthesized PCL was calculated by using the following equation:

(1) $$X_{c}\left(\%\right)=\frac{\Delta H_{m}}{\Delta H_{m}^{100}}\times 100$$

where Δ*Hm* is the enthalpy of fusion, and $\Delta H_{m}^{100}$ is the enthalpy of fusion of a 100% crystalline PCL.

By supposing a melting enthalpy of 148 kJmol^−1^ for the 100% crystallized PCL [26], the degree of crystallization for the synthesized PCL and for the grafted PCL chains was 52% and 37%, respectively (Table 1). The PLLA chains were amorphous.

**Figure 7:**
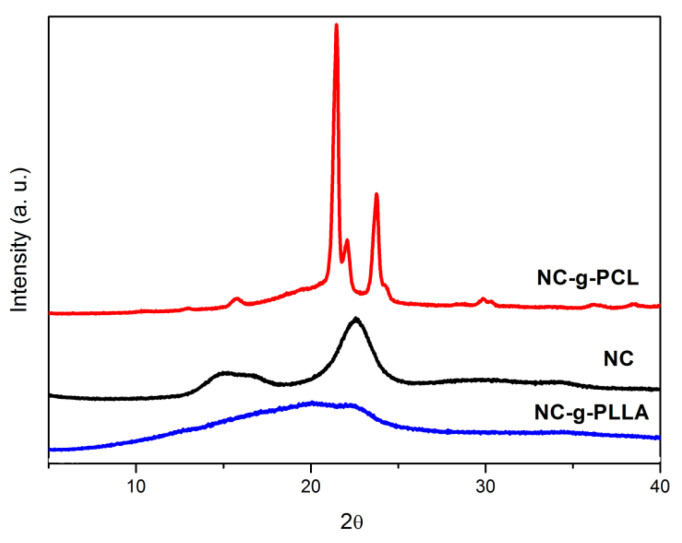
X-ray diffraction spectra for neat NC, NC-g-PCL, and NC-g-PLLA.

These results were confirmed by X-ray analysis, as reported in Figure 7. NC-g-PLLA showed an amorphous halo, whilst the NC-g-PCL diffraction pattern showed a combination of PCL crystallization peaks in the alpha form, at 21.4°, 22.0°, and 23.7°, and those of the cellulose nanocrystals at 14.9°, 16.6°, 22.4°, and 34.5° [31,32,33].

**Figure 8:**
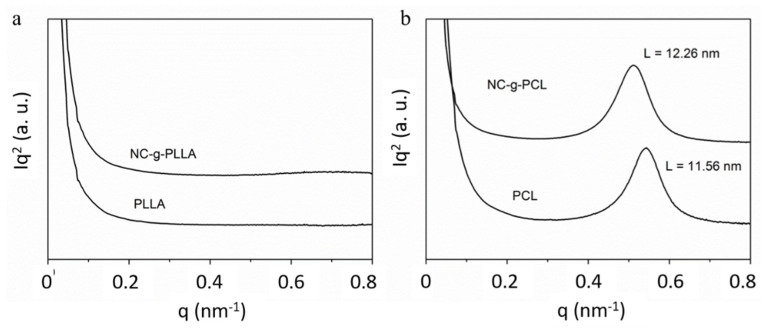
Iq2 vs. q for NC-g-PLLA (**a**) and NC-g-PCL (**b**).

The functionalized cellulose nanocrystals were also analyzed by SAXS experiments (Figure 8). NC-g-PLLA and PLLA did not show scattering, and therefore, no separated phases were detected. For both the NC-g-PCL and the synthesized PCL, a maximum in the scattered intensity was found, due to the difference in electronic density between PCL crystals and the rest of the material (NCs + amorphous PCL in NC-g-PCL and amorphous PCL for synthesized PCL). The calculated long period was slightly higher for NC-g-PCL.

**Figure 9:**
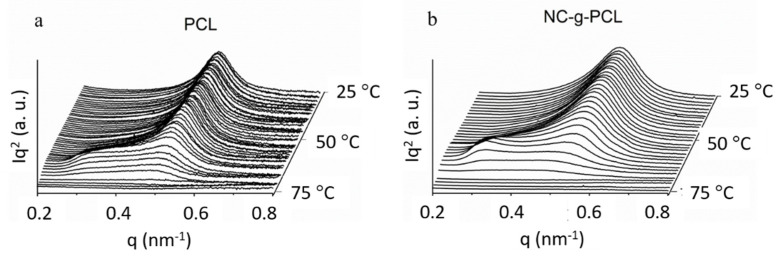
Scattering (SAXS) spectra with temperature for pristine PCL (**a**) and NC-g-PCL (**b**).

In Figure 9a,b, SAXS spectra at different temperatures for synthesized PCL and NC-g-PCL are reported. We obtained that the peak moved towards small angles and decreased in intensity with the increasing temperature, until its disappearance when PCL crystals melted, leaving a homogeneous material.

The melting temperatures obtained by SAXS were in agreement with the values obtained by DSC experiments.

## 4. Conclusions

In this work, a convenient “green” route to functionalize cellulose nanocrystals has been reported. *Yarrowia lipolytica lipase* was utilized as a biocatalyst for the functionalization of cellulose nanocrystals via a “grafting from” reaction using the hydroxyl groups in the surface of cellulose nanocrystals as initiators. ε-Caprolactone and L-lactide monomers were reacted by ring-opening polymerization to produce PCL- or PLLA-grafted nanocrystals, respectively. The grafting reaction was verified by using ^1^H-NMR, FT-IR, Raman spectroscopy, and TGA. In particular, we obtained that only the primary hydroxyl groups of the cellulose were able to start the ROP. However, in the case of NC-g-PLLA, we obtained that the primary hydroxyl group of cellulose was almost able to open the ring into two lactic acid monomers, thus stopping the reaction. For NC-g-PCL, only 1 in 5 cellulose primary hydroxyl groups were able to start the PCL polymerization, forming quite long PCL chains, indicating that in this case, the primary hydroxyl group of CL units is more reactive than those of cellulose. Furthermore, the amorphous and crystalline nature of the grafted polymeric chains was studied by DSC, WAXD, and SAXS experiments and correlated with their molecular weight.

## Figures and Tables

**Table 1:** Melting temperature (Tm) and degree of crystallinity (Xc) data obtained by DSC.

	Tm PCL (°C)	Xc PCL (%)
NC-g-PCL	50	37%
PCL	55	52%

## Data Availability

Not applicable.
